# First Detection of GES-5-Producing *Escherichia coli* from Livestock—An Increasing Diversity of Carbapenemases Recognized from German Pig Production

**DOI:** 10.3390/microorganisms8101593

**Published:** 2020-10-16

**Authors:** Alexandra Irrgang, Simon H. Tausch, Natalie Pauly, Mirjam Grobbel, Annemarie Kaesbohrer, Jens A. Hammerl

**Affiliations:** 1 Department Biological Safety, German Federal Institute for Risk Assessment (Bundesinstitut für Risikobewertung, BfR), D-10589 Berlin, Germany; simon.tausch@bfr.bund.de (S.H.T.); natalie.pauly@bfr.bund.de (N.P.); mirjam.grobbel@bfr.bund.de (M.G.); annemarie.kaesbohrer@bfr.bund.de (A.K.); jens-andre.hammerl@bfr.bund.de (J.A.H.); 2 Institute for Veterinary Public Health, University of Veterinary Medicine, 1210 Vienna, Austria

**Keywords:** carbapenemase, CPE, GES-5, GES-5B, whole-genome sequencing, livestock

## Abstract

Resistance to carbapenems due to carbapenemase-producing Enterobacteriaceae (CPE) is an increasing threat to human health worldwide. In recent years, CPE could be found only sporadically from livestock, but concern rose that livestock might become a reservoir for CPE. In 2019, the first GES carbapenemase-producing *Escherichia coli* from livestock was detected within the German national monitoring on antimicrobial resistance. The isolate was obtained from pig feces and was phenotypically resistant to meropenem and ertapenem. The isolate harbored three successive *bla*_GES_ genes encoding for GES-1, GES-5 and GES-5B in an incomplete class-I integron on a 12 kb plasmid (pEC19-AB02908; Acc. No. MT955355). The strain further encoded for virulence-associated genes typical for uropathogenic *E. coli*, which might hint at an increased pathogenic potential. The isolate produced the third carbapenemase detected from German livestock. The finding underlines the importance CPE monitoring and detailed characterization of new isolates.

## 1. Introduction

Carbapenemase-producing Enterobacteriaceae (CPE) are a global threat to human health. Carbapenemases are often associated with nosocomial infections (esp. KPC) but are also disseminated in the community [1]. While OXA-48, VIM, NDM and KPC carbapenemases are detected frequently from human infections in Germany, GES carbapenemase-producing bacteria were only isolated sporadically [2]. In 2018, *bla*_GES_ carbapenemase genes were recovered from 5 Enterobacteria and 14 *Pseudomonas aeruginosa* isolates from human infections, representing ~1% of detected CPE from human infections in Germany [2]. GES enzymes are serine proteases of the Ambler class A [3]. GES-1 was first described in *Klebsiella pneumoniae*. It exhibits strong activity against most β-lactams and results in an extended-spectrum β-lactamase (ESBL) phenotype [4]. Point mutations can lead to increased hydrolyzing activity and carbapenem substrate utilization (i.e., GES-5) [5].

While the carbapenemases VIM-1 and OXA-48 had been reported sporadically from German pig production in recent years [6,7], other carbapenemases have not been observed in German livestock so far. Here, we report the phenotypic and genotypic properties of the first GES-5-producing *E. coli* from livestock.

## 2. Materials and Methods

The *E. coli* isolate 19-AB02908 was obtained within the German national monitoring on antimicrobial resistance from a fecal sample of a fattening pig following the protocol of the European Reference Laboratory for Antimicrobial Resistance (EURL-AR) for selective CPE isolation (Available online: https://www.eurl-ar.eu/protocols.aspx). Phenotypic resistance was confirmed by determining minimal inhibitory concentration (MIC) values using the broth microdilution method according to CLSI guidelines (CLSI M07-A9). MIC values were interpreted according to EUCAST epidemiological cut-off values defined in 2013. Initial determination of the genotype was carried out by routine real-time PCR adapted from van der Zee et al. [8] and Swayne et al. [9], followed by diagnostic PCR for *bla*_GES_ (F: 5′-ATGCGCTTCATTCACGCAC-3′/R: 5′-TCCGTGCTCAGGATGAGTTG-3′) and subsequent Sanger sequencing of the PCR products. For genetic in-depth dissection, the isolate was subjected to long-read (Nanopore) and short-read (Illumina, PRJNA660949) sequencing. Hybrid assembly of the plasmid was carried out using Unicycler v.0.44 (provided by PATRIC 3.6.6). The complete plasmid sequence of pEC19-AB02908 is available under the GenBank Acc.No MT955355. Genome characterization was conducted with our in-house developed pipeline Bakcharak v1.0.0 (Available online: https://gitlab.com/bfr_bioinformatics/bakcharak) which implements ABRicate v1.0.1 (Available online: https://github.com/tseemann/abricate) for screening of antimicrobial resistance genes (using the NCBI amrfinder database [10]), plasmid markers (using the PlasmidFinder database [11]) and virulence factors (using the VFDB [12]). Plasmid contig identification was performed using platon (Available online: https://github.com/oschwengers/platon). Furthermore the MLST sequence type was inferred using mlst (Available online: https://github.com/tseemann/mlst) based on the pubmlst database [13].

## 3. Results

The isolate 19-AB02908 was obtained from a fecal sample of a fattening pig, which was taken in the course of the German resistance monitoring on CPE. Phenotypic antimicrobial resistances were detected for all tested penicillins and cephalosporins, as well as for tetracycline (MIC ≥ 64 mg/L), trimethoprim (MIC > 32 mg/L), and sulfamethoxazole (MIC > 1024 mg/L) (Table 1). The isolate further showed resistance to meropenem (MIC ≥ 0.25 mg/L) and ertapenem (MIC ≥ 0.25 mg/L), but only slightly reduced susceptibility to imipenem (MIC ≥ 0.5 mg/L).

**Figure 1:**
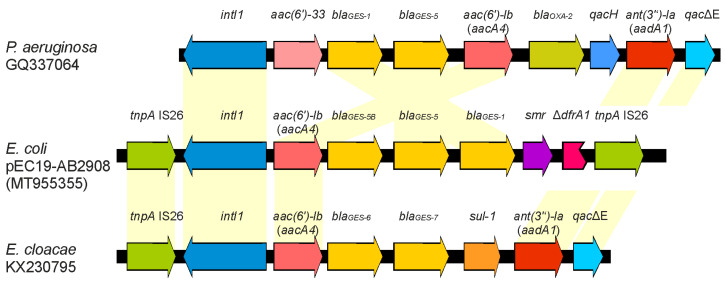
Comparison of the sequence organization of the *bla*_GES_ region with similar integrons available at GenBank [17,18]. The reading frames are presented with arrows; arrowheads indicate the direction of transcription.

The results of PCR sequencing indicated the presence of a GES-5 carbapenemase. Based on competing nucleotides at defined positions in the sequence chromatograms, the presence of up to two more GES variants seemed to be likely. The hybrid assembly of whole-genome sequencing revealed that there are three copies of the *bla*_GES_ genes separated by 157 bp intergenic regions on a 12 kb plasmid (GenBank Acc.No MT955355). The three-fold repetition of the *bla*_GES_ genes was verified by PCR using reverse complementary primers. As initially predicted, the prevailing *bla*_GES_ genes differed in one to two nucleotide positions from each other. One is the ESBL-encoding gene *bla*_GES-1_, followed by *bla*_GES-5_ and a second *bla*_GES-5_ with a silent mutation on nucleotide position 54 (G54A) (Figure 1). This gene variant was formerly named *bla*_GES-3_ (AY494717) [14], but was later renamed to *bla*_GES-5_ on the basis of its amino acid sequence relationship [15]. The current *bla*_GES-3_ gene (AB113580.1) was described by Wachino et al. [16]. We suggest naming the GES-5 variant with a silent mutation GES-5B according to the classifications of *bla*_TEM_ variants. Similar structures of class-I integrons with duplicated *bla*_GES_ variants have been found in *Pseudomonas aeruginosa* (GQ337064) and *Enterobacter cloacae* (KX230795) (Figure 1) [17,18].

Here, the unusual formation of three successive *bla*_GES_ variants was part of an incomplete integron, as the conserved CS3′ region was missing. In 2018, an incomplete class-1 integron with a quadruple of *bla*_GES-5_ gene cassettes was reported [19]. The authors suggested that the *bla*_GES_ gene cassettes tend to duplicate by site-specific recombination. Apart from antimicrobial resistances, the plasmid backbone is represented by a DNA region of 4.7 kb that can be found in several Enterobacteriaceae, like *Klebsiella pneumoniae* (e.g., LN824137) or *Salmonella* Typhimurium (e.g., CP050743). It is likely that a class-I integron with two GES variants has been integrated into this small plasmid. The presence of the third *bla*_GES_ gene might be a result of gene duplication by a recombination event in which the CS3′ site of the integron was deleted. Viedma et al. [18] suggested a direct repeat sequence (5′-ACAAA-3′) that might be involved in the gene duplication. In silico analysis revealed that this sequence is present 17 times on the pEC19-AB02908 plasmid, indicating that this recognition sequence might not be specific enough.

Besides the *bla*_GES_ carrying plasmid, the isolate harbored an additional 227 kb IncF plasmid conferring resistance to a variety of antimicrobials (aminoglycosides, beta-lactams, macrolides, and sulfonamides) as well as heavy metal resistance (Table 1). Therefore, a co-selection for the carbapenemase resistance can occur even if carbapenems or cephalosporins are not applied to the animals.

To determine a potential persistence of the plasmid in livestock, the farm was investigated comprehensively three months after the isolate was detected within the German national monitoring of antimicrobial resistance. Livestock and farm surrounding samples were taken and processed as described [20]. Additionally, real-time PCR from the enrichment cultures targeting the *bla*_GES_ gene was performed. However, no CPE could be detected in the samples indicating that a further spread of GES-producing enterobacteria did not occur.

Besides others, the isolate harbored a variety of virulence-associated genes typical for uropathogenic *E. coli* (i.e., *afaA*, *afaD*, *hylF*, *sfaX*, *iroN*, *iss*) suggesting an increased pathogenic potential [21]. This is untypical for phylogenetic group B1 as this phylogenetic group is often associated with high resistance while exhibiting only low virulence [22].

After VIM-1 and OXA-48, GES-5 is the third carbapenemase, which could be detected in German pig production. The entry source of these isolates remains speculative but the set of different carbapenemases found in livestock reflects the increasing diversity reported for CPE from human sources in Germany. The repeated detection of carbapenemases with presumed human origin in pig production shows that hygiene concepts should be followed. These are necessary not only to facilitate animal welfare, but also to prevent the transfer of zoonotic bacteria from humans to animals and vice versa. The newly reported isolate combines the zoonotic potential of a probable pathogenic *E. coli* with the limited therapeutic options due to its broad resistance features. This finding confirms the need for continuous monitoring in order to detect any spread of new resistance mechanisms in animal populations immediately.

## Figures and Tables

**Table 1:** Main characteristics of the GES-producing *E. coli* isolate 19-AB02908. Abbreviations of antimicrobials: AMP—ampicillin; ETP—ertapenem; FEP—cefepime, FOT—cefotaxime; FOX—cefoxitin; MERO—meropenem; SMX—sulfamethoxazole; TAZ—ceftazidime; TET—tetracycline; TMP—trimethoprim.

Phylogeny	Phenotypic Resistance	Resistance Genes	Plasmids	Virulence Associated Genes
**-ST1084** **-phylogenetic group B1**	AMP, ETP, FEP, FOT, FOX, MERO, SMX, TAZ, TET, TMP	*aac(6’)-Ib3*, *ant(3′’)-Ia*, *aph(3’’)-Ib*, *aph(6)-Id*, *bla*_GES-1_, *bla*_GES-5_, *bla*_GES-5B_, *bla*_TEM-1B_, *dfrA1*, *mph(B)*, *sul-1*, *sul-2*, *tet(A)*	12 kb (pEC19-AB02908) 227 kb	*afaA, afaD, cma, cvaC, hlyF, hra, iroN, iss, lpfA, ompT, sitA, terC, traT*
